# Draft genome sequence of *Modicisalibacter* sp. strain Wilcox isolated from produced water

**DOI:** 10.1128/mra.00990-24

**Published:** 2024-12-23

**Authors:** Damilare Ajagbe, William Marsh, Robert Murdoch, Babu Fathepure

**Affiliations:** 1 Department of Microbiology and Molecular Genetics, Oklahoma State University 7618 , Stillwater, Oklahoma, USA; 2 Battelle Memorial Institute, Biosciences Division 7618 , Columbus, Ohio, USA; SUNY College of Environmental Science and Forestry, Syracuse, New York, USA

**Keywords:** halophiles, produced water, *Modicisalibacter*, hydrocarbon degradation, metal tolerance, bioremediation

## Abstract

The draft genome sequence of *Modicisalibacter* sp. strain Wilcox, isolated from produced water, is presented. The genome is 3.6 Mb in size with 3,283 protein-coding genes, including genes for hydrocarbon degradation, heavy metal resistance, and adaptation to high salinity. This genome provides insights into molecular mechanisms of hydrocarbon degradation under hypersaline conditions.

## ANNOUNCEMENT

*Modicisalibacter* sp. strain Wilcox is a hydrocarbon-metabolizing, metal-resistant, halophilic bacterium isolated from an enrichment obtained using produced water (PW) collected from the First Wilcox formation (36.03561 N, 97.04483 W), Payne County, Oklahoma, USA (1). The PW had 4% (wt/vol) salinity, a pH of 7.42, and total dissolved solids of 146,600 mg/L, with several heavy metals in low concentrations. This aerobic bacterium, which can degrade benzene, toluene, ethylbenzene, xylene, and other hydrocarbons as carbon sources in the presence of high salinity (up to 4 M NaCl), was isolated and characterized as described previously (1). The isolate exhibited >99% 16S rRNA-gene identity to *Modicisalibacter tunisiensis* strain LIT2 in the family *Halomonadaceae*, which was isolated from an oil-field water in Tunisia (2, 3).

For DNA extraction, strain Wilcox was grown in serum bottles containing MSM with 1 M NaCl and 5 mM acetate at 35°C, and DNA was isolated using the DNeasy PowerSoil kit (Qiagen, Germantown, MD, USA), following the manufacturer’s instructions. For library preparation, the genomic DNA was randomly sheared into short fragments of 350 bp, and the DNA library was constructed using the NEBNext Ultra DNA Library Prep Kit (E3330L), New England Biolabs, Massachusetts, USA, following the manufacturer’s protocol. Sequencing was performed on the Illumina HiSeq 4000 platform by Novogene (Beijing, China) (1). The original data set contained 5,747,630 pairs of raw reads, each 150 base pairs long. A subset of 1.7 million read pairs was randomly sampled using the Seqtk v1.3 (4) to achieve a target genome coverage of approximately 100×. Reads were trimmed with Trimmomatic v0.39 (5), applying a sliding window of 4:30 and a minimum length threshold of 36 bp. Trimmed reads were assembled using SPAdes v3.13 (6), with a minimum contig size of 500 bp. Assembly quality was assessed using Quast v4.4 (7), and the final assembly consisted of 47 contigs with an N50 of 162,352 bp, an L50 of 8, a total length of 3,584,069 bp, and 67.25% G + C content. CheckM analysis v1.2.2 (8) indicated a genome completeness score of 99.92% and 1.04% contamination. Average nucleotide identity analysis using BLAST (ANIb) showed a 96.6% match with *Modicisalibacter tunisiensis* (GCA_020148005.1) (2).

Gene calling and annotation of the assembled genome were performed using IMG Annotation Pipeline v.5.0.19 (9). Genes involved in hydrocarbon degradation, adaptation to high salt, and heavy metal resistance were reviewed, and the metabolic pathways encoded were analyzed using KEGG orthology (KO), Pfam, and Clusters of Orthologous Genes assignments.

The isolation and sequencing of the genome of strain Wilcox adds to our repository of culturable extremophiles (10) and could provide insights into microbial growth and survival strategies in extreme environments characterized by high salinity, heavy metals, and hydrocarbons. Understanding the genomes of such organisms also helps develop bioremediation technologies for harsh environments.

## Data Availability

The assembly was submitted to the IMG database for gene calling, functional annotation, and to provide public access under genome ID number 2844586914. Unannotated scaffolds were deposited in NCBI GenBank under accession numbers WNXF00000000.1 and SRR10525457 for SRA reads. The NCBI RefSeq annotation is also available under ID GCF_010993675.1.
